# Ameliorative Potential of Hot Compress on Sciatic Nerve Pain in Chronic Constriction Injury-Induced Rat Model

**DOI:** 10.3389/fnsyn.2022.859278

**Published:** 2022-05-24

**Authors:** Kwan-Yu Chan, Wen-Ching Tsai, Chien-Yi Chiang, Meei-Ling Sheu, Chih-Yang Huang, Yi-Ching Tsai, Chia-Yun Tsai, Chia-Jung Lu, Zih-Ping Ho, De-Wei Lai

**Affiliations:** ^1^Department of Rehabilitation, Chang Bing Show Chwan Memorial Hospital, Changhua, Taiwan; ^2^Cardiovascular and Mitochondrial Related Disease Research Center, Hualien Tzu Chi Hospital, Buddhist Tzu Chi Medical Foundation, Hualien, Taiwan; ^3^Institute of Biomedical Sciences, National Chung Hsing University, Taichung, Taiwan; ^4^Rong Hsing Research Center for Translational Medicine, National Chung Hsing University, Taichung, Taiwan; ^5^Department of Medical Research, Taichung Veterans General Hospital, Taichung, Taiwan; ^6^Department of Medical Laboratory Science and Biotechnology, Asia University, Taichung, Taiwan; ^7^Graduate Institute of Biomedical Sciences, China Medical University, Taichung, Taiwan; ^8^Center of General Education, Buddhist Tzu Chi Medical Foundation, Tzu Chi University of Science and Technology, Hualien, Taiwan; ^9^Department of Medical Research, China Medical University Hospital, China Medical University, Taichung, Taiwan; ^10^Immunomedicine Group, Department of Molecular Biology and Cell Research, Chang Bing Show Chwan Memorial Hospital, Changhua, Taiwan; ^11^Experimental Animal Center, Department of Molecular Biology and Cell Research, Chang Bing Show Chwan Memorial Hospital, Changhua, Taiwan; ^12^Neurodiagnostic center, Chang Bing Show Chwan Memorial Hospital, Changhua, Taiwan; ^13^Department of Medical Research, Chang Bing Show Chwan Memorial Hospital, Changhua, Taiwan

**Keywords:** hot compress, chronic constriction injury, compound muscle action potential, synaptophysin, somatosensory cortex, hippocampus

## Abstract

Hot compress modalities are used to ameliorate pain despite prevalent confusion about which modality should be used and when. Most recommendations for hot compresses are based on empirical experience, with limited evidence to support its efficacy. To obtain insight into the nerve transmission mechanism of hot compresses and to identify the nerve injury marker proteins specifically associated with sciatic nerve pain, we established a rat model of chronic constriction injury (CCI) and performed mechanical allodynia, electrophysiology, and histopathological analysis. All CCI rats exhibited geometric representation of the affected hind paw, which indicated a hyper-impact on both mechanical gait and asymmetry of gait on day 28. The CCI model after 28 days of surgery significantly reduced compound muscle action potential (CMAP) amplitude, but also significantly reduced latency. Administration of hot compress for 3 weeks (heated at 40–42°C, cycle of 40 min, and rest for 20 min, three cycles each time, three times per week) significantly increased the paw withdrawal thresholds in response to stimulation by Von Frey fibers and reversed the CCI-induced reduced sciatic functional index (SFI) scores. Hot compress treatment in the CCI model improved CMAP amplitude and latency. The S100 protein expression level in the CCI+Hot compression group was 1.5-fold higher than in the CCI group; it dramatically reduced inflammation, such as tumor necrosis factor alpha and CD68 expression in nerve injury sites. Synaptophysin (Syn) expression in the CCI+Hot compression group was less than threefold in the CCI group at both nerve injury sites and brain (somatosensory cortex and hippocampus). This finding indicates that local nerve damage and inflammation cause significant alterations in the sensorimotor strip, and hot compress treatment could significantly ameliorate sciatic nerve pain by attenuating Syn and inflammatory factors from local pathological nerves to the brain. This study determines the potential efficacy and safety of hot compress, and may have important implications for its widespread use in sciatic nerve pain treatment.

## Introduction

Sciatic nerve pain affects a significant amount of the population worldwide. The common symptoms include widespread leg pain, paralysis, and related disabilities. Several synonyms exist for sciatica, such as lumbar radiculopathy, ischias, intervertebral disk disease, and nerve root entrapment. Patients usually receive treatment in primary care; however, a small percentage of the patients are referred to secondary care institutions and may eventually undergo surgery (Jacobs et al., 2011; Jensen et al., 2019). It is worth noting that approximately 90% of sciatica cases are caused by herniated discs and compression of the nerve root. Other causes include lumbar spinal stenosis and tumors (Valat et al., 2010). The common conservative treatments for sciatica include rehabilitation, massage, hot compresses, or corsets (including drug injection therapy) (Dahm et al., 2010; Dehghan and Farahbod, 2014; Lewis et al., 2015; Ropper and Zafonte, 2015). However, the molecular mechanisms underlying most conservative treatments remain unclear. Therefore, it is imperative to clarify sciatica with hot compress treatment and the molecular mechanisms involved in this research.

The conservative treatment of sciatica is mainly aimed at reducing pain through analgesics or reducing the nerve root pressure. Several studies have reported that conservative treatment may be limited in the natural course of sciatica or alleviating symptoms in some patients. Sciatica management includes drug and non-drug methods (Vroomen et al., 2000). The most common medications include non-steroidal anti-inflammatory drugs (NSAIDs) or acetaminophen. However, for some types of pain (e.g., acute low back pain), skeletal muscle relaxants/antispasticity drugs, antidepressants, corticosteroid injections (for back pain with radiculopathy), and opioids (for otherwise intractable pain) may be appropriate (Chou and Huffman, 2007; Pinto et al., 2012; Witenko et al., 2014; Machado et al., 2017). The non-pharmacological treatment strategy is to reduce the pain and related edema, while also promoting nerve repair to promote the restoration of normal function and activity (Jacobs et al., 2011; Savage et al., 2015). In this case, hot compresses are often used. However, involvement of the molecular mechanisms though moderate hot compress treatment conditions remain unclear.

Hot compresses may be associated with pain relief. Traditionally, a surface heat of 40–45°C treats the application site to a depth of approximately 1 cm; additionally, surface heating has been used in different forms (e.g., hot water bottles, towels, or bottles) to relieve menstrual cramps (Harris, 1938; Jo and Lee, 2018). Moreover, local hot compresses can promote blood circulation, eliminate local blood and body fluid retention, and reduce congestion and swelling, thereby reducing the pain caused by nerve compression (Petrofsky et al., 2013). In addition, synaptophysin (Syn) is widely distributed and plays a role in synaptic transmission, and may mediate the sciatic nerve pain induced by inflammation by acting on presynaptic Syn in the spinal cord dorsal horn neurons (Chou et al., 2002; Sommer and Kress, 2004; Zelenka et al., 2005). However, the molecular mechanisms involved in the regulation of hot compresses to reduce sciatic nerve pain are still not fully understood.

Our previous studies reported that chronic constriction injury (CCI) of the sciatic nerve model represents an advance in the study of neuropathic pain because the location of loose chromic gut ligatures on the rat sciatic nerve elicited behavior that seems analogous to that of humans with neuropathic pain (Chiang et al., 2014; Sheu et al., 2021). This study used the CCI rat model to explore information about hot compresses (non-pharmacological treatments) and the molecular mechanisms of nerve injury improvement. Furthermore, we determined the mechanism responsible for the effect of hot compress application in the CCI animal model by examining nerve repair and inflammation improvement, as well as the expression of S100, Syn, and TNFα at the injury site. The present study aimed to observe hot compress mediated treatment on injured hindlimbs following CCI sciatic nerve injury in a rat model to delineate the possible hot compress regulated mechanisms and therefore, determine the beneficial applications to patients with peripheral nerve injury.

## Materials and Methods

### Animals

In this study, 8–10 weeks old Sprague-Dawley rats weighing 250–300 g were kept in ventilated, humidity- and temperature-controlled rooms with a 12 h light/dark cycle. Soft bedding and sufficient food and water were provided. All the experiments were approved by the Animal Care and Use Committee of the Chang Bing Show Chwan Memorial Hospital. All efforts were made to minimize the number of animals used and their suffering.

### Animal Groups and Sciatic Nerve Surgery

The CCI surgery was modified from the description by Bennett and Xie’s (1988) CCI model. These animals were randomly assigned to five groups with a total of 40 animals, which were grouped into normal, sham, CCI, CCI+Hot compression, and hot compress alone groups, respectively. The CCI model was established in a manner similar to the method described by Chiang et al. (2014). Briefly, the right sciatic nerve was exposed and dissected from the surrounding connective tissue. Two ligatures of the 3-0 chromic gut were loosely ligated around the sciatic nerve without changing the morphology of the nerve. A sham surgery was performed by exposing the sciatic nerve without ligation. The surgical wound was closed in layers using 4-0 suture lines. All surgical procedures were performed under anesthesia with 4% isoflurane for induction, and 1–2% isoflurane for maintenance.

### Hot Compress Treatment

After 1 week of CCI surgery, the animals in the CCI+Hot compression, and hot compression groups were treated with a hot pack. These animals were anesthetized with 1% isoflurane, laid on a hot pack, and heated at 40–42°C with their right hind limb. The treatments were performed for a cycle of 40 min and rest for 20 min, three cycles each time, three times per week (execution begins at 1:00 p.m. on Monday, Wednesday, and Friday) for three consecutive weeks. The sham and CCI alone groups also performed the anesthesia process simultaneously.

### Electrophysiological Measurement

Electrical stimulation was performed using a Medelec Synergy electromyography (Oxford Instrument Medical Ltd., Surrey, United Kingdom). Three weeks after the hot compress treatment, all the animal groups were investigated for compound muscle action potential (CMAP) voltage before the animals were euthanized. After the animals were anesthetized with Zoletil 50 (40 mg/kg, i.p., Virbac), recording electrodes (Ambu A/S, Ballerup, Denmark) were fixed on the lateral and back sides, and real-time monitoring revealed that the R MEDIAN was a stable straight line before every operation. Electrical stimulation was performed through the right sciatic nerve.

### Mechanical Allodynia (Von Frey Test)

In this experiment, the animals were individually placed in small cages with frames and placed for 5 min prior to the test for adaptation. A Von Frey filament was applied to touch the hind paw ipsilaterally until it bent for 5 s. The filaments were applied range from 0.008 to 300 g; if the animal withdrew or shook the hind paws in at least four out of the five applications, it was considered as a positive response. The first step of “up-down” Von Frey method (Dixon, 1980; Deuis et al., 2017) is to estimate the response to filaments close to 50% withdrawal threshold. If there is no response, the next tested with a higher force filament; on the contrary, if there is positive response, the next tested with lower force filament. This continues until at four reads are archived after the first convert response. Calculation of the 50% threshold were detected though least six responses around the threshold.

### Sciatic Functional Index

The sciatic functional index (SFI) assessment method was modified from the description by Bain et al. (1989). Briefed method described following that a dark walking track of dimensions, 10 cm wide, 15 cm high, 50 cm long was prepared, and a paper strip of equal length and width was placed at the bottom of the track. The rat hind paws were soaked in red ink, and their hind footprints were clearly recorded. After the experiment, the strip was left to dry to measure the parameters. The factors for SFI included the print length (PL), toe spread (TS), and intermediary toe spread (ITS). The SFI value was calculated by the formula as follows: SFI = −38.3 (EPL−NPL)/NPL + 109.5(ETS−NTS)/NTS + 13.3(EIT−NIT)/ NIT−8.8. EPL, experimental print length; NPL, normal print length; ETS, experimental toe spread; NTS, normal toe spread; EIT, experimental intermediary toe spread; NIT, intermediary toe spread. SFI = 0 and −100 indicate normal and complete dysfunctions, respectively. All the groups were assessed four weeks after the hot compress treatment.

### Immunohistochemical Analysis

At day 28 after the hot compress treatment, the animals were anesthetized. The sciatic nerves and brain (brain cortex and hippocampus) were immediately removed, placed in 4% paraformaldehyde for 4 h, and then transferred to 30% sucrose at 4°C overnight. Each dissected tissue was embedded in a paraffin block and sectioned into 8 μ for sciatic nerves and 10 μm-thick sections for brain The tissue sections were deparaffinized and rehydrated through a series of xylene and ethanol washes, and antigen retrieval was performed in citrate buffer. The sections were then permeabilized with 0.2% Triton X-100 in PBS, blocked with PBS containing 1% bovine serum albumin (BSA), and stained with primary and secondary antibodies. These sections were subjected to immunohistochemical examination with anti-NGF (Chemicon, 1:250 dilution), anti-CD68 (Chemicon, 1:50 dilution), anti-TNFα (Abcam, 1:50 dilution), anti-synaptophysin (Abcam, 1:50 dilution), and anti-S100 (Thermo, 1:50 dilution) for the detection of inflammatory cells, inflammatory cytokines, small neurosecretory vesicles, and Schwann cells. Subsequently, the sections were washed and incubated overnight with secondary goat anti-mouse IgG-HRP (Thermo, 1:500 dilution). The staining was visualized using 3,3-diaminobenzidine (DAB). Analysis of IHC staining intensity using ImageJ, and selected highlight stained section tissues site were measured for each section of sciatic nerve (least 10 field/group). All experiments were performed least triplicates.

### Statistical Analysis

The quantification was done by assistant blind to the experimental condition. The data are expressed as mean ± SD. The statistical analysis was performed as described previously (Liu et al., 2021). Statistical comparisons between groups were made using two-way ANOVA (time × withdrawal threshold), with Bonferroni’s *post hoc* test for comparison between groups in Von Frey test model; statistical analysis on overall treatment effects compare with CCI were made by one-way ANOVA with Dunnett’s *post hoc* test. The statistical significance was set at a *p*-value < 0.05.

## Results

### Pain Hypersensitivity Induced by Chronic Constriction Injury Surgery

**FIGURE 1:**
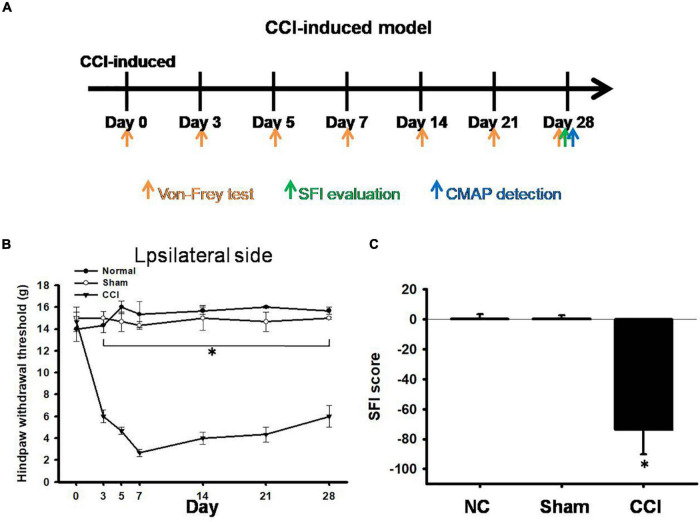
Pain hypersensitivity in chronic constriction injury (CCI) rats. **(A)** Schematic view of the establishment of neuropathic pain animal model. **(B)** Thresholds for hindpaw withdrawal responses to Von Frey filament stimulation on the ipsilateral site in the normal, sham, and CCI groups, two-way ANOVA with Bonferroni *post hoc* test. **(C)** Determination of the sciatic functional index (SFI) in the normal, sham, and CCI groups, the data was analyzed by one-way ANOVA with *post hoc* Dunnett’s test. ^∗^*p* < 0.001 was considered significant as compared with the CCI group, *n* = 8.

After CCI surgery, we examined the development of neuropathic pain-like behaviors using mechanical allodynia tests and evaluated the SFI. The pain behaviors were investigated on days 0 (the day of surgery) and 3, 5, 7, 14, 21, and 28 after CCI (Figure 1A). In our measurements obtained from CCI rats, the paw withdrawal threshold to mechanical stimuli was significantly reduced to 5.8 ± 0.4 g (*p* < 0.001) on day 3 and further reduced to 3.2 ± 0.5 g (*p* < 0.001) on day 7, compared with the pain threshold measured in the sham group (14.8 ± 0.8 g) (Figure 1B). In addition, all the CCI rats exhibited a geometric representation of the affected hind paw, which was indicated by a significantly lower SFI than that measured in the sham group or normal control on day 28 (Figure 1C). The pain had a hyper-impact on both mechanical and asymmetric gaits for at least 4 weeks after CCI (Figures 1B,C).

### Distal Digital Nerve Electrophysiology

**FIGURE 2:**
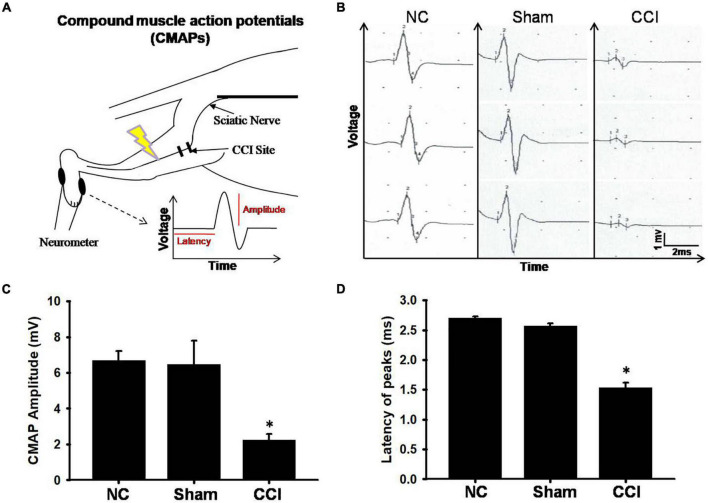
Electrophysiological findings for each group. **(A)** The schematic of electrode positions for electrophysiological recording neurometer segments of the digital nerve with respect to stimulation. **(B)** Representative spontaneous voltage recorded from involved plantar muscles from different groups. The scale bars represent 1 mV and 2 ms. **(C)** Amplitude and **(D)** latency of compound muscle action potential (CMAP). The vertical dash lines indicate the latencies for the marked amplitude (mV). The horizontal dash lines denote the latency time (ms) of activity in stimuli interval. Data were analyzed by one-way ANOVA with *post hoc* Dunnett’s test. ^∗^*p* < 0.001 was considered significant as compared with the sham group, *n* = 8.

The patterns of digital nerve activity varied as a function of the stimulus intensity and distal recording site (Figure 2A). The longest onset latency of the rats that responded to motor nerve stimulation was 10 ms; therefore, we set the onset latency of the rats that did not respond to the motor nerve stimulation at 10 ms. The intensity evaluated (20 ms, 5 mV) uniquely activated low-threshold axons, resulting in a composite response (Figure 2B). The CMAPs of the involved sciatic nerve were of attenuated amplitude of voltage and latency peak in the CCI groups (an amplitude of 1.5 ± 0.3 mV, latency 2.0 ± 0.08 ms) at 28 days post-injury in comparison with the sham groups (an amplitude of 6.4 ± 0.7 mV, latency 2.6 ± 0.05 ms), whereas no significant change was observed in the normal groups(an amplitude of 6.7 ± 0.5 mV, latency 2.7 ± 0.02 ms) (Figures 2C,D). Our data showed that the CCI model after 28 days of surgery not only showed a significant reduction in the CMAP amplitude, but also significantly reduced the latency.

### Effect of Hot Compress Treatment on Behavioral Parameters in Chronic Constriction Injury Induced Neuropathic Pain

**FIGURE 3:**
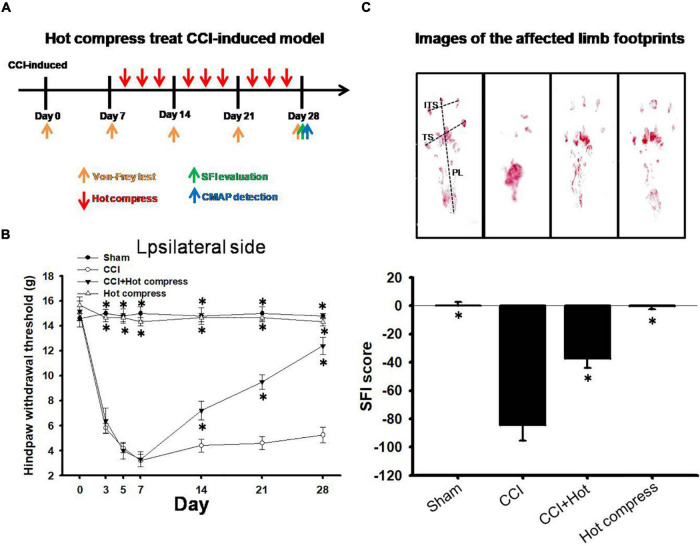
Hot compress may relieve CCI-induced neuropathic pain. **(A)** Schematic view of hot compress treatment process. **(B)** Thresholds for hindpaw withdrawal responses to the Von Frey filament stimulation on the ipsilateral site in sham, CCI, CCI + hot compress, and hot compress alone groups. Two-way ANOVA with Bonferroni *post hoc* test. **(C)** Footprints and determination of the sciatic functional index (SFI) in sham, CCI, CCI + hot compress, and hot compress alone groups, the data were analyzed by one-way ANOVA with *post hoc* Dunnett’s test. ^∗^*p* < 0.001 was considered significant as compared with the CCI group, *n* = 8. The factors for SFI included the print length (PL), toe spread (TS), and intermediary toe spread (ITS).

Behavioral changes, including mechanical allodynia, were significant in rats with CCI of the sciatic nerve by day 0, 3, 5, 7, 14, 21, and 28 compared to the sham groups. Additionally, the hot compress groups were treated on the 7th day after CCI surgery with a cycle of 40 min and rest for 20 min, three cycles each time, three times per week for three consecutive weeks, and the period of pain behavior was also investigated in the hot compress groups (Figure 3A). The administration of a hot compress cycle for 3 weeks significantly increased the paw withdrawal thresholds in response to stimulation by Von Frey fibers (Figure 3B) and reversed the CCI-induced reduction in the SFI scores (Figure 3C). The group treated with hot compress alone showed no mechanical allodynia or footprint deficits.

### Hot Compress Improved Electrophysiological Response After Chronic Constriction Injury Surgery

**FIGURE 4:**
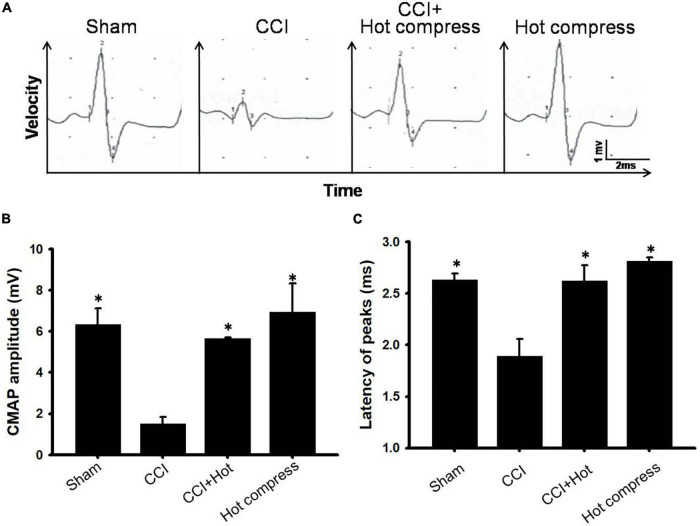
The effects of hot compress on a peak-to-peak amplitude and latency of the compound muscle action potentials (CMAPs) of the rat sciatic nerve. Panel **(A)** showed representative CMAPs tracings for sham, CCI, CCI+Hot compression, hot compress alone groups on the postoperative day 28. **(B)** The quantification of the hot compress effect on peak-to-peak amplitude and **(C)** latency is shown, respectively. Data were analyzed by one-way ANOVA with *post hoc* Dunnett’s test. ^∗^*p* < 0.001 was considered significant as compared with the CCI group, *n* = 8.

We also investigated the CMAP amplitude and latency. A lower CMAP amplitude has been previously associated with pain perception. To further confirm the associations between hot compression and neuropathic pain, we determined the CMAP amplitude and latency in the CCI model undergoing hot compress treatment (Figure 4A), which showed that the hot compress alone group did not show significant changes in the CMAP amplitude and latency. However, we found that the hot compress was successful in attenuating the CMAP amplitude and latency in the CCI model (Figures 4B,C). Therefore, we recognized that the hot compress treatment in the CCI model improved the CMAP amplitude and latency.

### Effect of Hot Compress on Histomorphological Alteration of Nerve System in Chronic Constriction Injury Model

**FIGURE 5:**
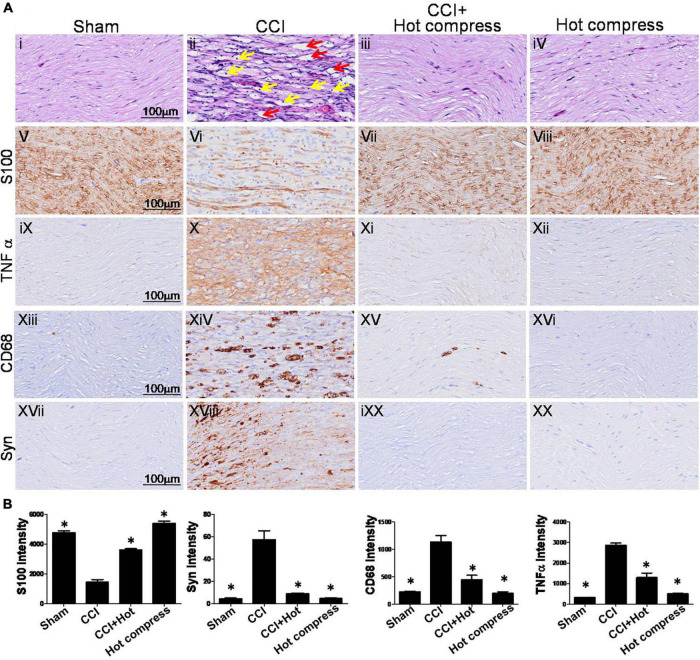
Histological examinations of rat sciatic nerve on the 28th day. **(A)** Through (i–iv) H&E staining and (v–xx) immuno-histopathological (IHC) staining over the distal end of the nerve in the different groups. Tissue sections from peripheral nervous were stained by IHC with anti-S100 antibody, which indicate location of Schwann cells and anti-CD68 antibody indicate microglia. (i) The sham group showed an organized cellular pattern without edema and infiltration. (ii) The CCI group showed worse cellular changes, the red arrow indicated cellular spaces, and the yellow arrow indicated inflammatory cell infiltration, (iii) the CCI + hot compress group showed minimal infiltration and cellular edema, (iv) the hot compress alone group not pathological effects. (v–viii) S100 and (xvii–xx) Syn immunoreactivity is seen for nerve injury markers in different groups and nerve pathology was confirmed. For (ix–xii) TNFα and (xiii–xvi) CD68, immunoreactivity is also seen localizing to the nerve inflammation dysphonic site. **(B)** A quantitative analysis of expression level of S100, Syn, TNFα, and CD68 in the different groups. Data were analyzed by one-way ANOVA with *post hoc* Dunnett’s test. ^∗^*p* < 0.001 was considered significant as compared with the CCI group, *n* = 8. Bar, 100 μm.

H&E staining of the sciatic nerve showed an organized cellular structure (Figures 5Ai–iv). In the CCI group, sciatic nerve injury was associated with various types of nerve damage as well as an increase in the cellular structure, infiltration, increase in intracellular spaces, and disorganized edema pattern because of the damage; the red arrow indicates cellular spaces and the yellow arrow indicates inflammatory cell infiltration (Figure 5Aii) compared with the sham group (Figure 5Ai). Treatment with hot compress reversed the constriction-induced pathological changes (Figure 5Aiii). The hot compress alone group showed no pathological cellular structures. In the sciatic nerve system, S100 protein is a valuable marker that can be used to identify myelinating Schwann cells, which is correlated with the expression of inflammatory cytokines and inflammatory cell deposition, such as TNFα and CD68. In this study, we found that the CCI group showed significantly reduced expression of S100 protein compared to the sham group. The S100 protein expression level in the CCI+Hot compression group was 1.5-fold higher than that in the CCI group (*F* = 139.6; df = 39, *p* < 0.001), and the hot compress alone group had no effect (Figures 5Av–viii). The trends of decreased S100 protein expression were reciprocal with increased TNFα expression and CD68 deposition. The TNFα expression level in the CCI group was more than fivefold of that of the sham group. In contrast, the CCI+Hot compression group showed significantly decreased TNFα expression compared to the CCI group (*F* = 113.5; df = 43, *p* < 0.001) (Figures 5Aix–xii). The CD68 expression level in the CCI group was twofold higher than that in the sham group. Additionally, the CCI + hot compression group showed a dramatic reduction in CD68 expression compared to the CCI group (*F* = 31.34; df = 46, *p* < 0.001) (Figures 5Axiii–xvi). No significant difference in the TNFα and CD68 expression was found in the hot compress alone group. Syn expression within the dorsal root ganglion is highly correlated with the severity of neuropathic pain. Syn was highly expressed in the CCI group and significantly increased compared with the sham, CCI+Hot compression, and hot compress alone groups (Figures 5Axvii–xx). The Syn expression level in the CCI+Hot compression group was less than threefold in the CCI group (*F* = 46.36; df = 41, *p* < 0.001). The quantitative expression of S100, Syn, TNFα, and CD68 in the different groups, respectively (Figure 5B).

### Effect of Hot Compress on Histomorphological Alteration of Hippocampus and Somatosensory Cortex in Chronic Constriction Injury Model

**FIGURE 6:**
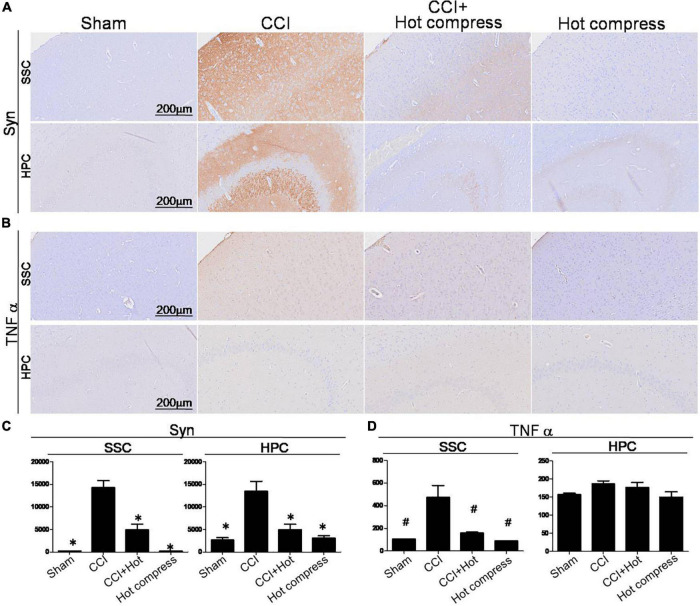
Histological examinations of Syn and TNFα expression over the SSC and HPC regions in the brain on the 28th day. IHC staining for **(A)** Syn and **(B)** TNF-α, **(A)** quantitative analysis of the expression level of Syn in the brain SSC (left) and HPC (right), and **(D)** quantitative analysis of the expression level of TNFα over the SSC (left) and HPC (right). Somatosensory cortex, SSC; hippocampus (CA3), HPC. Data were analyzed by one-way ANOVA with *post hoc* Dunnett’s test. ^∗^*p* < 0.001, ^#^*p* < 0.05 was considered significant as compared with the CCI group, *n* = 8. Bar, 100 μm.

**FIGURE 7:**
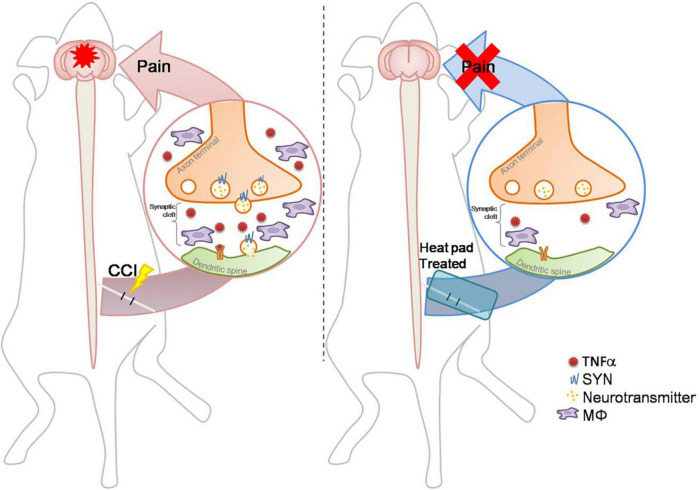
Schematic diagram: a conditional hot compress regulates the crosstalk between the inflammatory factors and synaptic transmission signals. The CCI-induced nerve injury increases TNFα and Syn levels and increases immune cell infiltration. The hot compress inhibits inflammatory factors and Syn to attenuate mechanical allodynia and abnormal electrophysiological responses, and exerts an effective analgesic effect on CCI-induced neuropathic pain through neuroendocrine cells to the brain mediation for inhibiting sciatic nerve pain.

Synaptophysin and TNFα expression within the brain hippocampus CA3 (HPC) and somatosensory cortex (SSC) reflect the brain plasticity response to CCI injury. In this study, decreased levels of Syn and TNFα expression were noted in the CCI + hot compression group than in the CCI group (Figures 6A,B). The synexpression levels in the HPC in the CCI group were threefold higher than those in the sham group and more than twofold higher than that in the CCI + hot compression group (*F* = 16.87; df = 43, *p* < 0.001), and SSC (*F* = 50.73; df = 43, *p* < 0.001). The TNFα expression level in the CCI group was twofold higher than that in the sham group and 1.7-fold higher than in the CCI + hot compression group (*F* = 13.49; df = 42, *p* < 0.05), respectively. Significant differences were found for TNFα expression within the SSC between the CCI and sham groups and between the CCI and CCI + hot compression groups. However, there was no significant difference in the expression of TNFα within the hippocampus in the CCI and sham groups and between the CCI and CCI + hot compression groups. Increased Syn expression within the sensorimotor strip indicates the responsiveness to peripheral nerve neuropathic injury. In this study, the increased intensity of evoked potential proportionally increased in the CCI group. The quantitative expression of Syn and TNFα in SSC and HPC from different groups are shown in Figures 6C,D. This finding indicates that local nerve damage and inflammation causes significant alterations in the sensorimotor strip, and hot compression treatment could significantly ameliorate the sciatic nerve pain by attenuating highly sensitive sensorimotor strips from local pathologic nerves (Figure 7).

## Discussion

Sciatic nerve pain is caused by a herniated disc and compression of the nerve root, leading to abnormal spontaneous and induced pain. Currently, the most conservative treatments for sciatica include rehabilitation, massage, hot compresses, and corsets (Kim et al., 2015). However, there is limited evidence to support the efficacy of specific modalities and the nerve transmission mechanisms involved in hot compression. In this study, we aimed to explore the effect of hot compression on neuropathic pain and its underlying mechanisms.

The exposure conditions are critical in hot compress treatments. Previous studies have reported that a heat exposure of >42°Cleads to nerve hyper-sensitivity through the release of CGRP in rat sciatic nerve axons in an *in vitro* model (Sauer et al., 2001; Ren et al., 2018). In addition, the axonal thermal responsiveness, when sensitized, also contributes to neuropathic pain following nerve injury or inflammation. Our data support that local hot compress (40–42°C) of the affected area 7 days after surgery in the CCI animal model can effectively reduce the inflammation and restore nerve sensitivity. In fact, some studies utilizing CCI models have found that the initial skin temperature of the affected limb initially increased in the course of 3–5 days, and finally decreased after 28–30 days (Wakisaka et al., 1991; Kim et al., 2012). Such findings have been attributed to autonomic mediation, such as denervation-induced supersensitivity to catecholamines and concomitant reduction of sympathetic vasoconstrictor outflow. Therefore, our study reported that moderate hot compression starting a week after the injury can effectively attenuate the infiltration of inflammatory factors in the affected area, which implies that periodic and continuous hot compression promotes vasoconstriction, and blood circulation contributes to nerve acceleration to the recovery phase. Furthermore, a recent study reported that a physical squeezing-induced nerve injury model produced large changes in the CMAP amplitude prior to large changes in the conduction velocity (Stecker et al., 2008; Fidancı and Öztürk, 2021). Therefore, we used CMAP electrophysiological testing to evaluate the therapeutic effects of hot compress treatment in the CCI model. This result shows that the CCI model reduced the CMAP amplitude and latency of time, while the hot compress treatment group effectively reversed the CMAP amplitude and latency of time.

Peripheral nerve pain is caused by the emergence and persistence of spontaneous activity of primary afferent neurons, which promotes the secondary and persistent increase in the excitability of the sensory circuit of the spinal dorsal horn, including sciatic nerve pain. In addition, especially long-term pain, the synthesis of more presynaptic vesicle protein release Syn in the nerve damage area will increase, which is related to the primary afferent C fibers (several of which are nociceptors) and the superficial spinal cord dorsal (Crair and Malenka, 1995; Sandkühler and Liu, 1998). The excitatory synaptic transmission between angular neurons is involved, and is a key factor for the long-term enhancement of synapses at thalamocortical synapses (Chou et al., 2002; Chen et al., 2009). In our study, we found that the hot compress treatment group can effectively reduce the expression of Syn in the nerve compression area or in the cerebral cortex area, which implies that hot compression attenuated sciatic nerve pain through reduced Syn. Furthermore, the expression of S100 proteins is observed to occur mostly in the Schwann cells in the peripheral nervous system. S-100 is one of the most useful markers for definitively characterizing nerve damage. Our results showed that hot compression can effectively restore the nerve cells at the location of the nerve injury, including increasing the expression of S100; thus, we cannot exclude the possibility that it can restore the injured nerve microenvironment under moderate hot compress treatment conditions, including microglia cells and Schwann cells.

Proinflammatory cytokines, including TNFα and IL-1β induce pain, and their inhibitors that relieve pain have been extensively researched (Schäfers and Sommer, 2007). The development of hyperalgesia despite the loosely placed ligature around the sciatic nerve in the CCI model without actual mechanical damage indicates that the development of neuropathic pain is mainly associated with inflammation and the released mediators, rather than nerve injury. Additionally, peripheral nerve injury also leads to the activation of peripheral (Schwann cells) and central (microglia and astrocyte) glial cells and begins to secrete proinflammatory cytokines, including TNFα, IL-1β, and PGE2 (Zelenka et al., 2005). These positive feedback loops between inflammatory mediators and glial activation and between glia and neurons lead to the further enhancement and maintenance of neuropathic pain (Ignatowski et al., 1999). In our current study, we demonstrated that moderate hot compresses can dramatically reduce inflammatory factors, both in the location of the nerve injury and the brain. Moreover, the pain relief effect of hot compresses may be enhanced by increasing the blood circulation and reducing the inflammatory factors. However, we also found that the macrophage marker protein CD68 is also significantly reduced, which implies that the hot compress treatment may also partially regulate switching of the M1/M2 phenotype of macrophages and act as a trigger or accelerator of auxiliary tissue repair (Nakagawa et al., 2018), which is a topic worthy of further elucidation. Endogenous tissue repair and regeneration in the nerve injury that critical step is to managing inflammatory response (Franco and Fernández-Suárez, 2015; Wynn and Vannella, 2016; Li et al., 2018). In the brain, the somatosensory cortex and hippocampus are responsible for the registration of physical pain (Yang and Chang, 2019). In particular, neurogenesis contributes to learning and memory and may trigger the development of chronic pain (Apkarian et al., 2016). Some studies have reported that the levels of biologically active TNFα are increased in the locus coeruleus and hippocampus at day 8 arrival maximum and following recovery to normal, which corresponds to the development of hyperalgesia in the CCI model (Covey et al., 2000). This is consistent with the results of our study. However, the CCI model showed higher TNFα expression in the somatosensory cortex than the sham group, while the hot compress treatment relieved TNFα expression in the CCI model. These results imply that the hot compress treatment on the affected area not only reduces inflammation in the affected area, but also effectively reduces the inflammatory factors secreted to the brain. Taken together, we recognized that moderate hot compresses may effectively improve sciatic nerve pain and inhibit inflammation caused by Syn and immune cell infiltration. Moreover, the scope of hot compress treatment is not only regulated by the affected area; however, it is also closely related to the plasticity of the brain through nerve conduction.

## Conclusion

In summary, we demonstrate the potential efficacy and safety of hot compresses in the treatment of sciatic nerve pain, and confirmed that hot compresses may significantly improve sciatic nerve pain by reducing Syn and inflammatory factors from local pathological nerves to the brain. This target regulatory molecular pathway may enhance our understanding of the mechanisms related to hot compress therapy, which may help to use hot compress therapy in a wider range of neuropain diseases and have important significance in assisting different neuropathic therapies.

## Data Availability Statement

The original contributions presented in the study are included in the article/supplementary material, further inquiries can be directed to the corresponding author.

## Ethics Statement

The animal study was reviewed and approved by the Chang Bing Show Chwan Memorial Hospital.

## Author Contributions

K-YC, W-CT, C-YC, and D-WL conceived and designed the experiments. C-JL and C-YH supported CMAP technical assistance. Y-CT, C-YT, and Z-PH performed most of the experiments and analyzed the data. K-YC, M-LS, and D-WL made intellectual contributions and contributed to the writing and revision of the manuscript. All authors agreed to be accountable for all aspects of the work, ensuring integrity and accuracy.

## Conflict of Interest

The authors declare that the research was conducted in the absence of any commercial or financial relationships that could be construed as a potential conflict of interest.

## Publisher’s Note

All claims expressed in this article are solely those of the authors and do not necessarily represent those of their affiliated organizations, or those of the publisher, the editors and the reviewers. Any product that may be evaluated in this article, or claim that may be made by its manufacturer, is not guaranteed or endorsed by the publisher.
